# A Method for Dynamically Selecting the Best Frequency Hopping Technique in Industrial Wireless Sensor Network Applications

**DOI:** 10.3390/s18020657

**Published:** 2018-02-23

**Authors:** Erlantz Fernández de Gorostiza, Jorge Berzosa, Jon Mabe, Roberto Cortiñas

**Affiliations:** 1 Electronics and Communications Unit, IK4-Tekniker, Calle Iñaki Goenaga 5, 20600 Eibar, Spain; jorge.berzosa@tekniker.es (J.B.); jon.mabe@tekniker.es (J.M.); 2 Computer Science Faculty, University of the Basque Country UPV/EHU, Paseo M. Lardizábal 1, 20018 Donostia-San Sebastián, Spain; roberto.cortinas@ehu.eus

**Keywords:** wireless sensor networks, robustness, coexistence mechanisms, interference avoidance, security, frequency hopping, channel characterization

## Abstract

Industrial wireless applications often share the communication channel with other wireless technologies and communication protocols. This coexistence produces interferences and transmission errors which require appropriate mechanisms to manage retransmissions. Nevertheless, these mechanisms increase the network latency and overhead due to the retransmissions. Thus, the loss of data packets and the measures to handle them produce an undesirable drop in the QoS and hinder the overall robustness and energy efficiency of the network. Interference avoidance mechanisms, such as frequency hopping techniques, reduce the need for retransmissions due to interferences but they are often tailored to specific scenarios and are not easily adapted to other use cases. On the other hand, the total absence of interference avoidance mechanisms introduces a security risk because the communication channel may be intentionally attacked and interfered with to hinder or totally block it. In this paper we propose a method for supporting the design of communication solutions under dynamic channel interference conditions and we implement dynamic management policies for frequency hopping technique and channel selection at runtime. The method considers several standard frequency hopping techniques and quality metrics, and the quality and status of the available frequency channels to propose the best combined solution to minimize the side effects of interferences. A simulation tool has been developed and used in this work to validate the method.

## 1. Introduction

Wireless Sensor Networks (WSN) are one of the industrial applications that benefit the most from the license-free nature of the Industrial, Scientific and Medical (ISM) band. Nevertheless, the ISM band has to be shared with other devices and systems using standard communication protocols such as Wireless Local Area Network (WLAN) or Bluetooth [1]. This situation leads to interferences in the communication channel and, as a result, produces (pseudo-) random transmission errors. Re-transmitting interfered packets might eventually succeed, but at the expense of increased latency and energy consumption of the devices.

Lost packets and increased latency directly affect the QoS of the network, first by the direct loss of arbitrary packets and second by the side effects of the missed packets such as breaking a multi message or state-full process that has to be started from the beginning (i.e., pairing, network establishment, discovery, etc.) [2].

Additionally, not all interferences are unintentionally produced by coexisting networks. External attackers may try to block the communication channel in order to perform a Denial of Service (DoS) [3] attack. DoS security attacks are specifically designed for interfering with the communication link, either hindering or totally blocking the communication channel. Thus, communication protocols that do not appropriately manage the communication channel may not be able to provide a satisfactory security level.

All in all, interference avoidance mechanisms that reduce the need for retransmissions are highly desired in WSN domains in order to minimize energy consumption, reduce unnecessary degradation of QoS and reliability as well as to raise security. 

**Figure 1:**
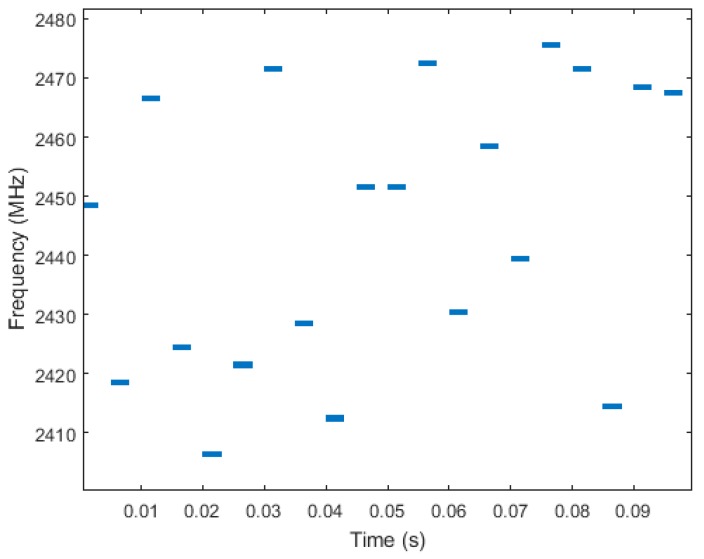
Example of a Frequency Hopping schedule.

In this paper we present a method for supporting the design of communication solutions for saturated and dynamic environments from the point of view of channel interference. This method characterizes frequency hopping techniques based on the quality of the available frequency channels. Frequency hopping techniques relay on the use of multiple frequency channels over time as opposed to statically allocating and using a single frequency channel. The main idea is that, in case of channel interference, frequency hopping helps in minimizing the side effects by avoiding (at least temporally) the interfered frequency. Figure 1 shows a frequency hopping schedule example in which multiple frequency channels are selected at different time instants.

The core of the method is based on several standard frequency hopping techniques as well as quality metrics for the characterization of available and valid frequency channels. The proposed method homogenizes the notation used to describe the different techniques and normalizes the different quality metrics based on the statistical properties of the RSSI values. Thanks to the homogenization and normalization, the different techniques can be fairly compared based on quality and performance indicators. Additionally, the method is flexible enough to accommodate new frequency hopping techniques, quality metrics and performance indicator approaches. Thus, it is scalable and extensible with new additions.

All the techniques and metrics are combined into a two-dimensional matrix. Each technique and metric pair represents a possible solution which provides a subset of selected or preferred channels and the sequence in which the channels are used. For some techniques, this sequence is purely random while for others the sequence will change based on specific parameters. For instance, in techniques based on probabilistic channel usage, there are channels within the subset which are used more often because they are considered to be of “better” quality. On the other hand, and regarding quality metrics, some metrics are better suited for characterizing static interferences while others are better used for dynamic interferences that hop between channels.

All the solutions from the matrix are compared to determine the best one: the solution that results in the fewer number of interferences for the given interference range, either produced by coexisting networks or by external factors (such as malicious attacks).

The proposed method can be used in two complementary scenarios: (a) as a deployment planning supporting tool in order to decide on the best frequency channels, channel quality metrics and frequency hopping techniques and (b) as a runtime management component for the dynamic allocation of communication frequency channel. In the former case, a network topology can be planned based on the frequency channel subset, channel hop sequence and the number of hops of each node to the gateway. In the latter case, the management component can be implemented into a device which will monitor the quality of the environment using the selected quality metric and dynamically adapt to the environment conditions by switching to the most suitable frequency hopping technique. Additionally, the method can also take into account the characteristics of constrained systems (either computationally or energetically) in order to select the most suitable solution.

To the best knowledge of the authors, this is the first attempt to define a method that homogenizes, combines and compares several frequency-hopping techniques for network deployment and real-time adaptation. As will be shown in Section 3, current approaches compare either different frequency hopping techniques or characterization metrics, but no common comparison is made to specify the best solution taking into account frequency channel quality behaviour and its evolution over time. No technique found in the SoTA adapts the frequency hopping technique dynamically to the environment conditions. Several frequency hopping techniques are analysed, but their use is not modified at run time (nor the quality metric) if the interferences pattern (and frequency channel quality) changes in the medium-long term.

This paper only considers the theoretic validation of the proposed method in simulated scenarios. The method has been implemented as a Matlab tool, which has been used in this work for testing and validation purposes. The validation considers realistic network coexistence and interference scenarios and applies the method to compute the best solution for the given scenario, evaluating frequency channel interference in terms of Packet Error Rate (PER). Thus, the Matlab tool is used as a deployment planning supporting tool and its efficiency is evaluated.

The paper is structured as follows: Section 2 presents a summary of the current interference avoidance mechanisms used in standard communication protocols, followed by Section 3 which includes the related work for this paper. Section 4 summarizes the metrics used by the method to characterize and determine the quality of the available frequency channels. Section 5 introduces the different frequency hopping techniques considered by the method. Section 6 describes the core of the method and Section 7 presents the evaluation. Finally, Section 8 summarizes and concludes the paper pointing out future steps.

## 2. Interference Avoidance in Standard Protocols

Within the 2.4 GHz ISM band, different standards have been developed to enable interoperability between devices. Each of these standards uses a different mechanism for interference avoidance. The well-known and widely used Wireless Local Area Network (WLAN), for instance, adopts the IEEE 802.11 standard [4] which makes use of Direct Sequence Spread Spectrum (DSSS) [5]. In DSSS the message signal is modulated with a bit sequence known as Pseudo Noise (PN) that consists of pulses of a much shorter duration (larger bandwidth) than the pulse duration of the message signal. The same PN code is used by the receiver to reconstruct the message signal. The modulation of the signal makes the resulting signal noisier and, thus, more immune to unintentional or intentional interference. WLAN divides the 2.4 GHz ISM band into 11 channels with 22 MHz bandwidth each. Thus, only three channels can be used at the same time avoiding overlapping of the frequency bands. DSSS is not suitable to low-power systems due to the high data rates involved.

The IEEE 802.15 standard [6] is the base for Wireless Personal Area Networks (WPAN). Here, the range is smaller than for the WLAN, but the energy consumption is considerably reduced. The first sub-standard IEEE 802.15.1 is known as Bluetooth [7], which uses Frequency Hopping Spread Spectrum (FHSS) [5]. In FHSS the message signal is transmitted by rapidly switching among many frequency channels, using a pseudorandom sequence known to both transmitter and receiver. FHSS divides the available frequency band into sub-bands or channels, and hops among them in a predetermined order. Bluetooth divides the 2.4 GHz ISM band into 79 channels with 1MHz bandwidth each, and hops from channel to channel up to 1600 times per second. 

Even if Bluetooth is more energetically efficient than WLAN, it is not suitable for some applications where autonomous operation of battery-powered devices is desirable. The sub-standard for Low-Rate Wireless Personal Area Networks (LR-WPAN) is IEEE 802.15.4 [8], which divides the spectrum into 16 non-overlapping channels (starting from channel 11 to channel 26) with a channel width of 3 MHz.

The manifold benefits of wireless technologies, specially the absence of cables, make Wireless Sensor Networks (WSN) attractive for industrial applications as well. However, the adoption of wireless technologies in industry poses extra challenges, mainly because factory environments are typically harsh in terms of interferences, noise and physical obstacles. Several industrial organizations, such as ZigBee [9], HART [10] and ISA [11] have been promoting the use the IEEE 802.15.4 standard to introduce wireless technologies in industrial applications. While ZigBee only utilizes DSSS provided by the IEEE 802.15.4 physical layer, WirelessHART [10] and ISA100 [12] adopt channel hopping and channel blacklisting to improve the data transmission reliability [13]. In ZigBee, all the transmissions stay on the same channel unless the entire network decides to hop to another channel.

WirelessHART uses only 15 of the 16 channels defined by the IEEE 802.15.4: channels 11 to 25. Channel 26 is not included as the corresponding frequency is not allowed in some countries [14]. Communication among network devices is arbitrated using Time Division Multiple Access (TDMA) [15], which allows for the scheduling of link activity. To enhance reliability, TDMA is combined with channel hopping mechanisms. WirelessHART employs non-adaptive frequency hopping where each link in the network switches randomly between the 15 available channels. Moreover, channels subject to interference may be eliminated due to blacklisting, and so, the number of available channels may be less than 15. Transmissions are synchronized in 10 ms timeslots and during each timeslot all available channels can be used simultaneously by the various nodes in the network. This allows 15 packets to be propagated through the network at the same time while minimizing the risk of collisions.

Like WirelessHART, ISA100 uses TDMA along with collision avoidance mechanisms, such as Carrier Sense Multiple Access with Collision Avoidance (CSMA-CA) and Clear Channel Assessment (CCA) [16], resulting in increased data transmission reliability. Before packets are transmitted, the transmitter listens on the channel on which it intends to transmit in order to assess if the channel is clear. If the channel is not clear, the transmitter backs off for a random amount of time, after which it attempts to retransmit the packet. Devices communicate according to various channel hopping techniques, with each subsequent transmission utilizing the next channel defined in the hopping sequence. Different devices use different offsets in the hopping sequence, resulting in interleaved hopping of devices operating in a wireless subnet. Every data packet needs to be acknowledged by the receiving device, and unacknowledged packets are retransmitted on a different channel over a different frequency. Hopping techniques include channel blacklisting and adaptive hopping. Channel blacklisting is a centralized decision made by the system manager, while adaptive hopping is a localized decision based on statistics of wireless parameters collected by each device. Adaptive hopping allows wireless devices to adapt their hopping sequences based on the quality of communication with specific neighbours.

## 3. Related Work

There is a significant amount of literature that contains studies of the performance and coexistence of the different IEEE standards. The authors in [17], for instance, examine the reliability of a point to point communication with a real IEEE 802.15.4 hardware by measuring the Packet Error Rate (PER) and the Received Signal Strength Indicator (RSSI), both in indoor and outdoor RF environments. The obtained results are used to calibrate the error model in the ns-2 network simulator in order to produce a more real simulation environment and evaluate the IEEE 802.15.4 network performance in a more reliable way. Furthermore, the coexistence between IEEE 802.15.4 and IEEE 802.11 networks is addressed, measuring the impact these two wireless technologies have on each other when operating concurrently and in range. It is concluded that the IEEE 802.15.4 network operation has practically no negative influence on a concurrent IEEE 802.11 communication, but if no care is taken about the operational channels of the two technologies, the IEEE 802.11 network will have a negative effect on the performance of the IEEE 802.15.4 network. From the performed measurements, there should be at least 7 MHz offset between the operational frequencies for a satisfactory performance of the IEEE 802.15.4 network.

In [1], the coexistence between IEEE 802.11, IEEE 802.15.1 and IEEE 802.15.4 is examined through mathematical analysis. For different combinations of affected and interfering networks, the PER caused by cross-technology interference is calculated. This PER is calculated from the Signal to Noise Ratio (SNR) at the affected wireless network receiver, and it is evaluated in terms of distance between interfering and affected networks, packet interval and channel separation. When varying the interferer distance from the receiver or the packet interval, the transmitter and interferer channels are chosen as follows: IEEE 802.11 on channel 1 (2412 MHz), IEEE 802.15.1 on channel 3 (2410 MHz) and IEEE 802.15.4 on channel 12 (2410 MHz), thus constituting co-channel interference. When varying the channel separation, the interferer channel separation from the transmitter is varied from −15 to 15 MHz, thus constituting adjacent channel interference. It is concluded that the IEEE 802.15.1 is more affected by IEEE 802.15.4 interference than vice versa. On the other hand, the IEEE 802.15.1 results to be more resistant than IEEE 802.15.4 against IEEE 802.11 interference.

Nevertheless, these studies do not deepen in interference avoidance mechanisms such as frequency hopping. Some other work can be found in the literature related to models and simulators of frequency hopping systems [18,19,20]. The authors in 18 modelled a frequency hopping wireless communication system based on the Signal Processing Worksystem (SPW) developed by CoWare (acquired by Synosys in 2010) [21]. The designed system is tested in terms of its Bit Error Rate (BER) performance under broadband and partial-band noise. The interfering noise is generated and modelled as the sum of a broadband Additive White Gaussian Noise (AWGN) and a multi-tone interference noise, where the jammer spreads some interference signals on a number of discrete frequency points. The system defines 32 frequency channels, and the frequency hopping pattern is generated according to a certain pseudo-random algorithm.

In [19], a simulator is designed to simulate and evaluate a frequency hopping spread spectrum communication systems using VisSim Comm software [22], which is mainly designed to simulate and analyse communication systems. The presented simulator is capable of simulating a complete system (transmitter, receiver and medium) operating under a noisy environment. The carrier frequencies for the FHSS system are varied in a pseudorandom manner within a wideband channel. The performance of the communication system is evaluated in terms of BER, and it is concluded that the FHSS significantly reduces the probability of error of a system operating under narrow band jamming conditions.

In [20], mathematical modelling is used to simulate and analyse the performance improvement using frequency hopping spread spectrum with popular modulation techniques using Matlab-Simulink. The baseband signal is combined with a randomly generated carrier frequency. The carrier frequency is controlled by a Pseudorandom Noise (PN) sequence generator and the signal is transmitted over AWGN. The received data is demodulated using the same PN code, and compared to the original input data to calculate the BER.

Theses simulators deal only with frequency hopping systems with pseudorandom frequency sequences, and do not consider adaptive frequency hopping techniques, where the selected frequency channels are adapted to the conditions and characteristics of the environment interferences. The method presented here takes into consideration different hopping techniques and channel quality metrics found in the literature. These hopping techniques and metrics are interlaced to analyse different possible solutions and obtain the best subset of channels as well as a predetermined hopping sequence for a given scenario where the network under study has to coexist with unwanted interfering networks.

## 4. Channel Characterization Metrics

This section describes the different channel characterization metrics taken into consideration in the analysis. Different quality metrics can be used in order to classify frequency channels based on interference level. Regardless of the metric used, the method must classify lowly interfered channels as “good” and highly interfered channels as “bad”. The Received Signal Strength Indicator (RSSI), for instance, measures the RF power level received by an antenna. The channel is characterized by its centre frequency and bandwidth. The higher the RSSI signal, the worse the channel, as it is highly interfered by unwanted coexisting systems. RSSI is usually measured in dBm units, that is, the power ratio (*P*) in decibels (dB) referred to one milliwatt (mW):

(1) $$P\left(\mathrm{dBm}\right)=10\cdot \log_{10}\frac{P\left(\mathrm{mW}\right)}{1\;\mathrm{mW}}$$

The RF power level of a radio signal ranges between 0 dBm and −120 dBm. The closer this value is to −120 dBm, the better, because that means there is little to no interference. Typical lowly interfered environments range between −100 dBm and −80 dBm.

Alternatively, frequency channels can be classified using Packet Error Rate (PER), which represents the rate of non-received to sent frames. A channel is classified as bad if its PER exceeds the system defined threshold. Bit Error Rate (BER) can be used in a similar way. 

**Figure 2:**
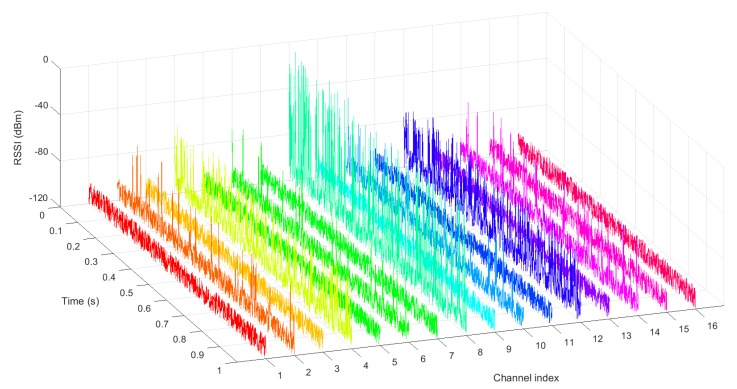
Example of Received Signal Strength Indicator (RSSI) signal over 1 s for an IEEE 802.15.4 network with 16 channels. The RSSI values lie within −120 dBm and 0 dBm. Higher RSSI values indicate channels interfered to a greater extent, while lower RSSI values indicate channels interfered to a lesser extent.

In the following sections we will focus on the RSSI as a channel quality indicator, as the measurements and calculations involved with RSSI are less complicated, and RSSI values are easily available from the chipsets. However, the work presented here is equally applicable to other indicators (such as PER) with little or no modification. RSSI values must be measured over a specified observation time for all the available channel frequencies. Figure 2 shows an illustrative example of RSSI signals over an observation time of 1 s for the 16 channels available in an 802.15.4 network. On purpose, some channels are affected only by white noise, while others are interfered to a greater or lesser extent by some coexisting systems.

From RSSI values, different statistical properties can be obtained for channel characterization [23]. These statistical properties are calculated over a specified observation time for every available frequency channel.

- Mean value (mean): A low mean value of the RSSI signal over the observation time indicates low RF power in the channel and, thus, a lowly interfered channel. The lower the mean value, the better the channel.
- Standard deviation (std): A high standard deviation indicates a highly interfered channel because of the high variability of the RF power. The lower the standard deviation, the better the channel.
- Skewness (skew): The skewness indicates the asymmetry of the RSSI distribution. A symmetric distribution, which means skewness of around zero, is expected when the RSSI signal is only affected by white noise and no interfering radio transmissions. When multiple RSSI peaks occur due to radio transmissions, the distribution of the RSSI signal extends to higher values and, thus, a higher value of skewness is expected. The skewness of a signal, *x*, is calculated by Equation (2), where *n* is the number of RSSI samples, *mean*(*x*) the mean value and *std*(*x*) the standard deviation of *x*.
  (2) $$skew\left(x\right)=\frac{1}{n}\frac{\sum_{i=1}^{n}{\left(x_{i}-mean\left(x\right)\right)}^{3}}{std{\left(x\right)}^{3}}$$
- *x*% quantile (quan): The *x*% quantile of a set of samples describes the cut value which divides the statistical distribution so that *x*% of the samples are below the cut value. A simple way to calculate the quantile is to sort the RSSI values and find the entry with the index corresponding to the *x*% of the vector length. The lower this value, the less peaks occur in the RSSI signal and the lower interference from other radio systems is expected.
- Number of samples over threshold (soth): It is the number of RSSI samples over a specified power level threshold. The lower this value, the less peaks occur in the RSSI signal.

All mentioned properties have in common that lower values indicate less interference and thus better channels. These properties must be transformed into a channel gain metric, *H*, that is directly proportional to the channel quality, so that it can be directly introduced in the equations presented in Section 5. To do this, a simple linear adjustment is performed:

(3) H=α·x+β

where *x* is an arbitrary statistical property of the RSSI signal and *α* and *β* are parameters to be adjusted. Furthermore, the *H* metric is normalized between [0, 1], so that the *α* and *β* parameters are derived in the following way:

(4) $$\left[\min\left(x\right),\max\left(x\right)\right]\to \left[1,\;0\right]\;\begin{array}{c} 1=\alpha \cdot \min\left(x\right)+\beta \\ 0=\alpha \cdot \max\left(x\right)+\beta \end{array}\}\to \{\begin{array}{c} \alpha =\frac{1}{\min\left(x\right)-\max\left(x\right)}\\ \beta =-\frac{\max\left(x\right)}{\min\left(x\right)-max\left(x\right)} \end{array}$$

**Figure 3:**
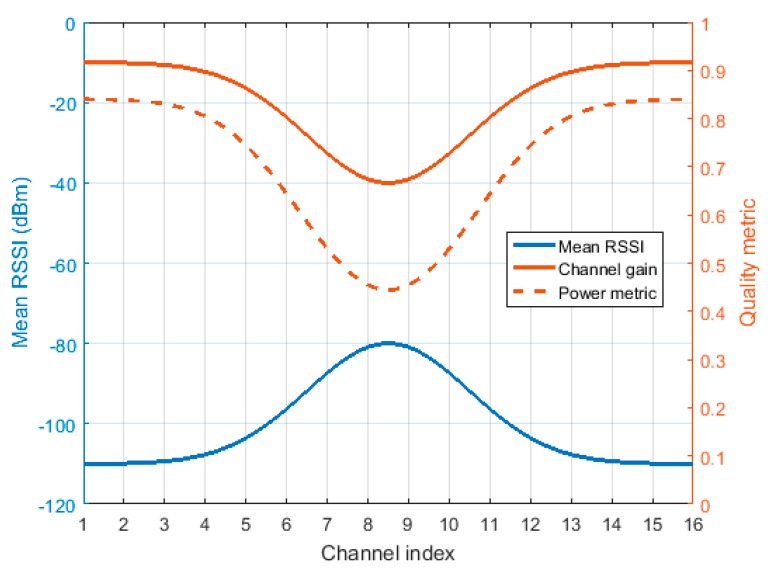
Example of linear projection from the mean value of the RSSI signal (blue) to a normalized channel gain, *H* (red). The dashed red line represents the power metric, *Q*, derived from the channel gain.

Figure 3 shows an illustrative example of this linear projection from the mean value of the RSSI signal (that ranges from −120 dBm to 0 dBm) to a normalized quality metric. The power metric presented in Equation (5), *Q* = |*H*|^2^, is also represented.

**Figure 4:**
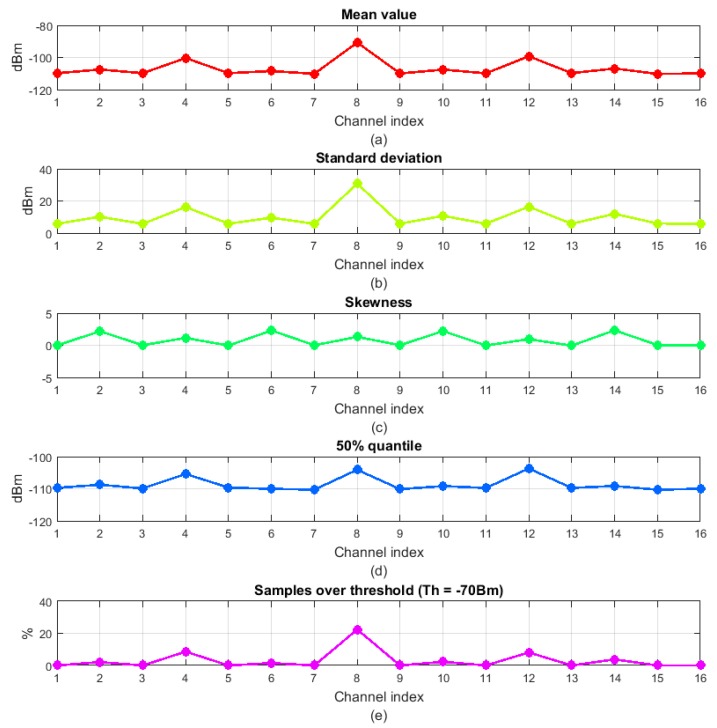
Different statistical properties obtained from the RSSI values in Figure 2: (**a**) Mean value; (**b**) Standard deviation; (**c**) Skewness; (**d**) 50% quantile; (**e**) SOTH with −70 dBm threshold.

**Figure 5:**
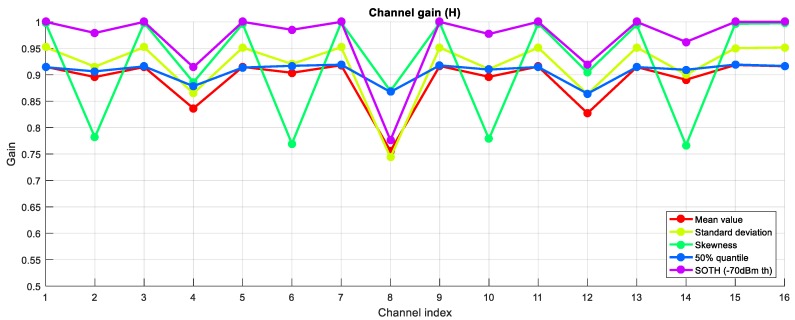
Normalized channel gains from RSSI statistical properties of Figure 4.

For the RSSI values in Figure 2, the calculated statistical properties are represented in Figure 4. The derived channel gains are represented in Figure 5. 

It can be observed that most of the statistical properties result in similar channel gains for the considered RSSI signal. This does not stand for the skewness metric, which suggests that channels 2, 6, 10 and 14 are worse than channels 4, 8 and 12, for instance. Even if channels 2, 6, 10 and 14 have higher interfering power peaks, they also have more continued traffic, resulting in a more symmetric skewness function and, thus, lower skew values. Therefore, the skewness metric would be a better indicator of channel quality when dealing with dynamic interferences that vary a lot in time, while the others would be better when dealing with static interferences that hold in time.

So, one of the open questions will be to find the most appropriate metric for a reliable channel quality indicator. Even more, for some metrics, some parameters will have to be adapted for each specific application (the cut value and the power threshold for the x% quantile and SOTH respectively). One of the main objectives of the proposed method is to automatically decide the best RSSI property for each specific scenario.

## 5. Frequency Hopping Techniques 

**Figure 6:**
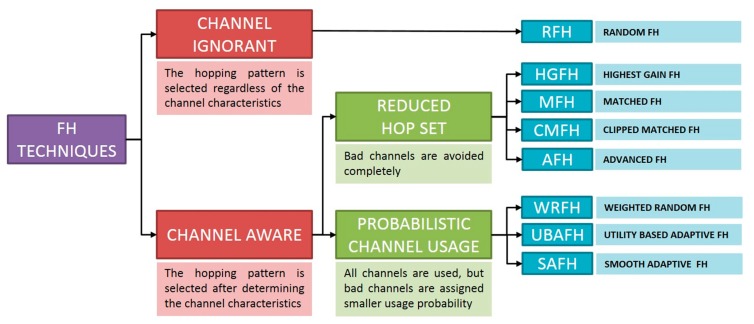
Classification of frequency hopping techniques.

This section describes the different frequency hopping techniques taken into consideration in the analysis. Hopping techniques determine not only the subset of channels, but also the way these channels are scheduled. Frequency hopping techniques can be classified as either channel-ignorant or channel-aware [24]. With channel-ignorant frequency hopping techniques, the hopping pattern is selected regardless of the channel characteristics, whereas with channel-aware frequency hopping techniques, the hopping pattern is selected after determining the channel characteristics. Channel-aware hopping techniques can be further classified as reduced-hop-set and probabilistic-channel-usage techniques [24,25,26]. Algorithms based on reduced hop sets totally avoid bad channels. On the other hand, algorithms based on probabilistic channel usage use all channels, although bad channels are assigned a smaller usage probability that depends on environmental conditions. Figure 6 summarizes the frequency hopping technique types. These techniques are explained in more detail in the following subsections.

### 5.1. Channel-Ignorant Techniques

#### Random Frequency Hopping

In Random Frequency Hopping (RFH) [27], the available frequency band is divided into *K* narrow sub-bands, and transmission is carried out by transmitting short bursts of data on one sub-band at a time, hopping from sub-band to sub-band in a pseudo-random way.

With channel ignorant techniques such as RFH, over a sufficiently large amount of time, all sub-bands will be used a roughly equal number of times, and so, transmission over bad sub-bands is inevitable. This will cause system performance degradation resulting in high packet error rates. To solve this problem, channel-aware frequency hopping techniques are proposed, where the hopping pattern is selected after determining the channel characteristics. Channel-aware or adaptive frequency hopping algorithms are able to provide better throughput against static interferences, dynamic interferences or both static and dynamic interferences, but they might be more vulnerable to jamming attacks as they decrease the frequency diversity.

### 5.2. Channel-Aware, Reduced-Hop-Set Techniques

Frequency hopping techniques based on reduced hop sets select *M* channels from the available *K* channels, and hop among them in a pseudo-random manner.

#### 5.2.1. Highest Gain Frequency Hopping

The most obvious example of the reduced-hop-set techniques is the Highest Gain Frequency Hopping (HGFH), where the selected *M* channels are the ones with highest gain. 

#### 5.2.2. Matched Frequency Hopping

Channels with high gains tend to be adjacent to each other, and so, it might be easier for a jammer to jam adjacent frequency channels. The Matched Frequency Hopping (MFH) not only selects channels with high gains, but also tries to select channels with dispersed frequencies [28]. In this technique, the main parameter used to select the *M* channels is the set of channel gains, *H_k_*, from which the power metrics, *Q_k_*, are calculated:

(5) $$Q_{k}={\left|H_{k}\right|}^{2}$$

To select channels with high gains and reasonably well-spaced frequencies, a normalized power metric, *B_k_*, and a cumulative metric, *C_k_*, are defined:

(6) $$B_{k}=\frac{Q_{k}}{\sum_{i=1}^{K}Q_{i}}\;k\in \left\{1,2,\ldots ,K\right\}$$

(7) $$C_{k}=\sum_{i=1}^{k}B_{i}$$

The cumulative metric will always be a monotonically increasing function of *k*. This metric is used with the *M* equally spaced values over [0, 1) given by

(8) $$y_{m}=\frac{1}{M}\left(m-\frac{1}{2}\right)\;m\in \left\{1,2,\ldots ,M\right\}$$

to determine the indices of the selected channels. The index of the *m*th channel is given by the value of *k* such that

(9) $$C_{k-1}\leq y_{m}<C_{k}$$

**Figure 7:**
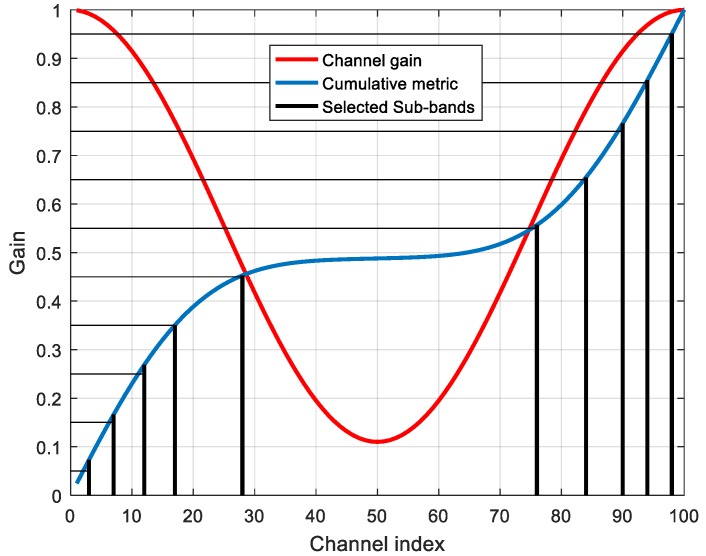
Channel selection for Matched Frequency Hopping (MFH) technique. The red line represents the channel gain and the blue line represents the cumulative sum of the power metric. The selected channels are represented as vertical black lines. The best channels are selected from the intersections between the cumulative metric and the equally spaced horizontal lines.

Figure 7 illustrates how *M* = 10 channels are selected among *K* = 100 available channels using this method. For the considered metric, using the highest gain technique would result in selecting the first five and last five channels, while only attending to channel separation would result in equally spaced channels. The MFH algorithm tends to select channels with high gains while avoiding selecting clusters of adjacent channels.

#### 5.2.3. Clipped Matched Frequency Hopping

The Clipped Matched Frequency Hopping (CMFH) is an evolution of the MFH technique [29]. The channel gains are first clipped at a certain threshold, proportional to the maximum gain, and then, the clipped channel gains are decreased by this threshold. That means any channel gain less than or equal to the threshold, *ξ*max(|*H_k_*|^2^), is set to zero. The power metrics, *Q_k_*, are redefined so that

(10) $$Q_{k}=\{\begin{array}{ll} {\left|H_{k}\right|}^{2}-\xi \max({\left|H_{k}\right|}^{2}), & {\left|H_{k}\right|}^{2}>\xi \max({\left|H_{k}\right|}^{2})\\ 0, & {\left|H_{k}\right|}^{2}\leq \xi \max({\left|H_{k}\right|}^{2}) \end{array}$$

The MFH technique is then applied to the new clipped power metrics, instead of using directly |*H_k_*|^2^ as it is done in the MFH technique. The CMFH technique will result in a hopping set with more concentrated channels than in the MFH technique, but with higher gains.

#### 5.2.4. Advanced Frequency Hopping

The Advanced Frequency Hopping (AFH) technique further improves the performance of the MFH technique by selecting more channels with higher gains [30]. The modified power metrics are given by

(11) $$Q_{k}=\frac{{\left|H_{k}\right|}^{2}}{\left(1+\alpha \right)max\left({\left|H_{k}\right|}^{2}\right)-{\left|H_{k}\right|}^{2}}$$

where *α* is a small scaling factor that can be adjusted to obtain different responses. The MFH technique is then applied to the new power metric.

**Figure 8:**
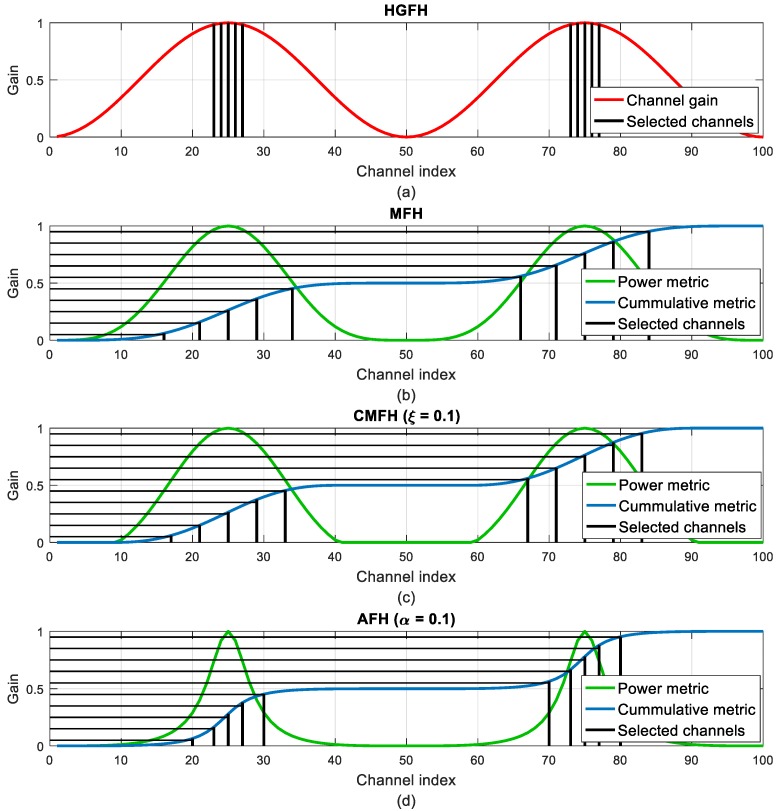
Comparison of channel selection between different frequency hopping techniques: (**a**) Highest Gain Frequency Hopping (HGFH); (**b**) Matched Frequency Hopping (MFH); (**c**) Clipped Matched Frequency Hopping (CMFH) with *ξ* = 0.1; (**d**) Advanced Frequency Hopping (AFH) with *α* = 0.1. The selected channels are represented as vertical black lines. HGFH directly uses the channel gain (red) to select the best channels, while MFH, CMFH and AFH use the cumulative metrics (blue) obtained from the power metrics (green) and their intersections with the equally spaced horizontal lines.

Figure 8 illustrates the channel selection for the HGFH, MFH, CMFH and AFH techniques. In all the cases *M* = 10 channels have been selected. It can be seen how the MFH technique selects more dispersed channels compared to the HGFH. The CMFH and AFH techniques modify the power metric to cluster the channel selection around the highest gain channels but still preserving certain channel separation.

**Figure 9:**
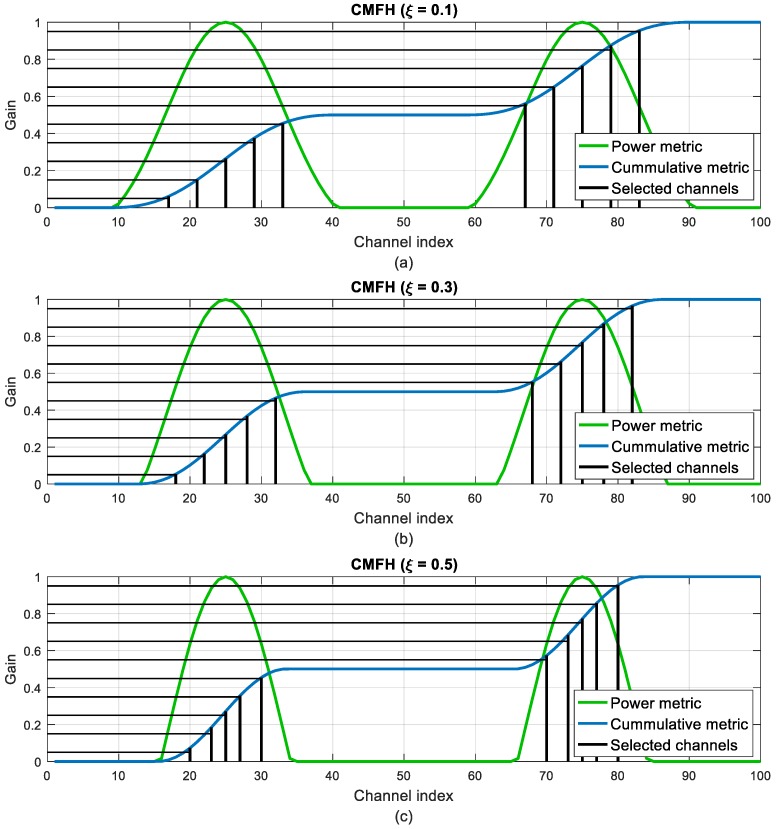
Channel selection evolution in the Clipped Matched Frequency Hopping (CMFH) technique when varying the threshold parameter: (**a**) *ξ* = 0.1; (**b**) *ξ* = 0.3; (**c**) *ξ* = 0.5.

**Figure 10:**
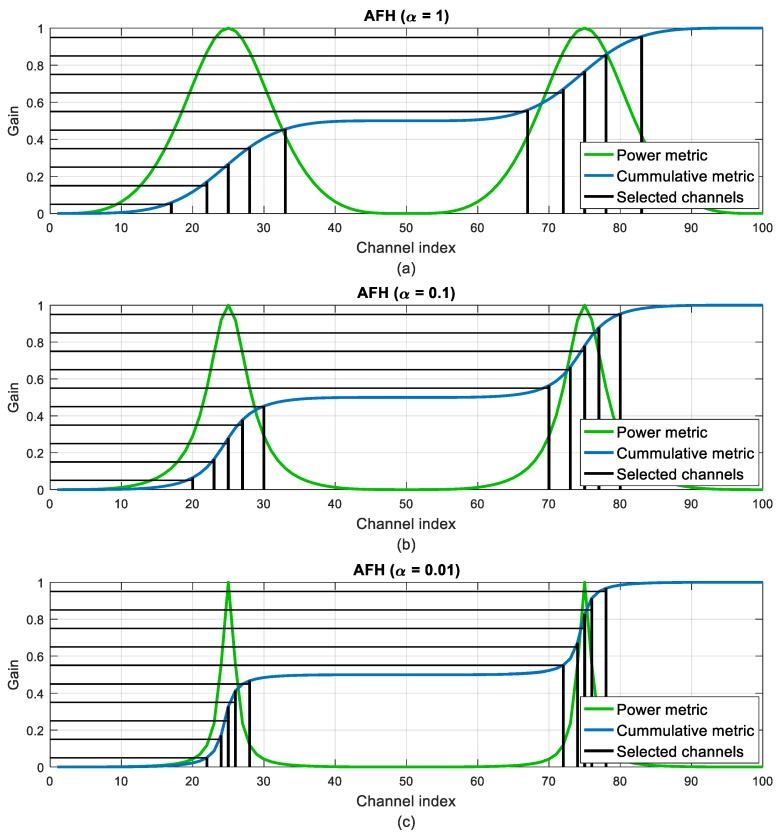
Channel selection evolution in the Advanced Frequency Hopping (AFH) technique when varying the α parameter: (**a**) *α* = 1; (**b**) *α* = 0.1; (**c**) *α* = 0.01.

Figure 9 and Figure 10 illustrate the evolution of the channel selection in the CMFH and AFH techniques when varying the *ξ* and *α* parameters. When increasing the threshold in the CMFH technique, the selected channels tend to concentrate around the highest gain channels, as more bad channels are discarded. The same happens when decreasing the *α* parameter in the AFH technique.

### 5.3. Channel Aware, Probabilistic-Channel-Usage Techniques

Unlike the reduced-hop-set techniques, probabilistic-channel-usage techniques use all the available channels, but channels marked as good are used with higher probability.

#### 5.3.1. Weighted Random Frequency Hopping

In the Weighted Random Frequency Hopping (WRFH) technique, the hopping set contains all of the *K* channels, just like with the standard RFH, but when deciding the hopping sequence, a non-uniform probability distribution is used so that channels with higher gains are more likely to be selected [24]. The probability, *P_k_*, of selecting channel *k* is given by

(12) $$P_{k}=\frac{{\left|H_{k}\right|}^{2}}{\sum_{i=1}^{K}{\left|H_{i}\right|}^{2}}\;k\in \left\{1,2,\ldots ,K\right\}$$

where *H_k_* is the same channel gain introduced in Equation (5). This equation is analogous to the normalized power metric in the MFH technique (Equation (6)). For each hop, the selection of the channel is carried out by a cumulative metric, *C_k_*, as it is done with the MFH technique:

(13) $$C_{k}=\sum_{i=1}^{k}P_{i}$$

The index of the *n*th channel is given by the value of *k* such that

(14) $$C_{k-1}\leq y_{n}<C_{k}\;n\in \left\{1,2,\ldots ,K\right\}$$

where *y_n_* is a pseudo-random number over [0,1). This is the main difference with the MFH technique, as the corresponding *y_m_* numbers in the MFH were *M* equally spaced values over [0,1). So, in the WRFH technique, all the channels can be selected with more or less probability, while in the MFH technique, there were only *M* selected channels that would be used with equal probability.

**Figure 11:**
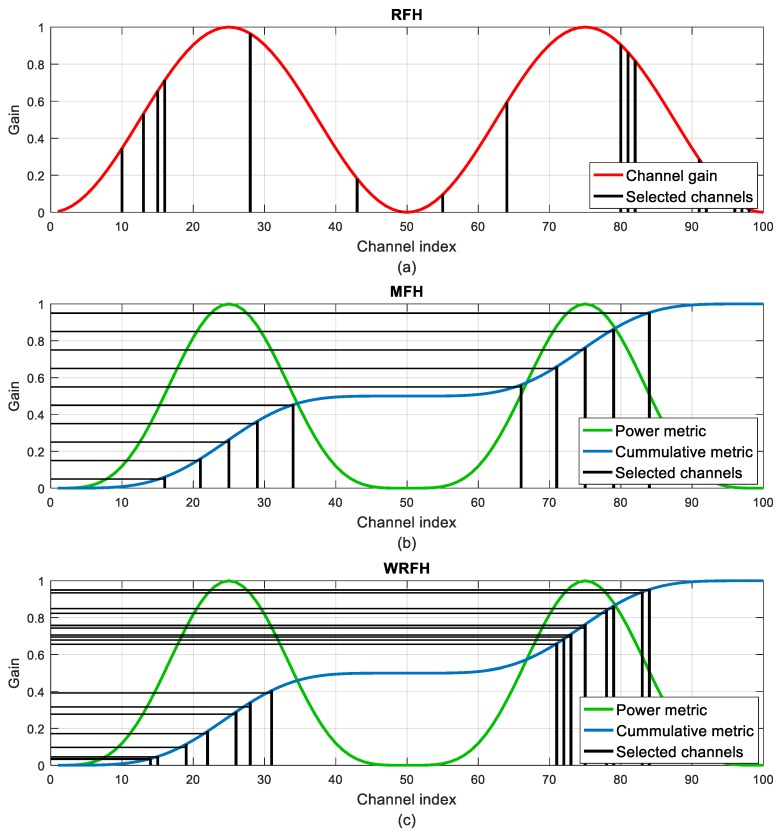
Comparison of channel selection between different frequency hopping techniques: (**a**) Random Frequency Hopping (RFH); (**b**) Matched Frequency Hopping (MFH); (**c**) Weighted Random Frequency Hopping (WRFH). In the RFH and MFH cases, horizontal lines are equally spaced while in the WRFH case, horizontal lines are randomly spaced.

Figure 11 illustrates the differences between the RFH, MFH and WRFH techniques. As an example, 20 frequency hops have been considered for the three hopping techniques. In the case of the random and weighted random techniques, this results in 20 different channels, whereas in the MFH technique, some or all of the selected 10 best channels have to be repeated. Because of its random behaviour, the RFH technique selects some channels with a very low gain. This is partially avoided by the WRFH as the cumulative metric around the lowest gain channels is hardly increased and is less likely to be intercepted by the horizontal lines representing the *y_n_* random values. However, as the number of channel hops approaches infinite, it would be inevitable to hop over the channels with the least gain, which does not happen with the MFH technique.

#### 5.3.2. Utility Based Adaptive Frequency Hopping

The Utility Based Adaptive Frequency Hopping (UBAFH) technique extends the performance of the WRFH by introducing a parameter, *α*, which is called temperature [31]:

(15) $$P_{k}=\frac{Q_{k}^{\alpha }}{\sum_{i=1}^{K}Q_{i}^{\alpha }}$$

The parameter *α* can be adjusted to achieve different behaviours. Low temperatures will result in almost even channel usage, while for high temperature values, only the best channels will be used. When *α* = 1, the probability distribution happens to be the same as for the WRFH technique. Additionally, lower and upper bounds on the usage probability can be introduced so that

(16) $$P_{min}\leq P_{k}\leq P_{min}$$

*P_min_* is introduced to ensure a minimum degree of frequency diversity, while *P_max_* prevents the algorithm from converging to scenarios where only a few channels are used.

#### 5.3.3. Smooth Adaptive Frequency Hopping

Smooth Adaptive Frequency Hopping (SAFH) assigns usage probabilities to all channels based on an exponential smoothing filter for the quality metric to estimate and predict the channel conditions [32]. The algorithm uses exponential smoothing to predict the quality metric of the next time step. The predicted power metrics are referred as $Q_{k}^{\prime }$, while the measured power metric is referred to as *Q_k_*. The predicted power metric at time (*t* + 1) is calculated by using the predicted metric at time *t* plus an adjustment for the error that occurred in the last forecast (measured power metric minus predicted power metric):

(17) $$Q_{k}^{\prime }\left(t+1\right)=Q_{k}^{\prime }\left(t\right)+\alpha \cdot \left(Q_{k}\left(t\right)-Q_{k}^{\prime }\left(t\right)\right)$$

(18) $$\{\begin{array}{c} Q_{k}^{\prime }\left(t+1\right)=\alpha \cdot Q_{k}\left(t\right)+\left(1-\alpha \right)\cdot Q_{k}^{\prime }\left(t\right)\\ Q_{k}^{\prime }\left(1\right)=Q_{k}\left(0\right)\to \left(initial\;condition\right) \end{array}$$

The balance between new and old data is controlled by the smoothing factor *α* in the range between [0,1]. When *α* approaches 1, the filter gives more weight to recent data and has less of a smoothing effect. When *α* approaches 0, the effect of current observation is ignored and only the smoothed past is retained. By recurrent substitution,

(19) $$Q_{k}^{\prime }\left(t+1\right)=\alpha \cdot \left[\sum_{j=0}^{t-1}{\left(1-\alpha \right)}^{j}\right]\cdot Q_{k}\left(t-j\right)+{\left(1-\alpha \right)}^{t}\cdot Q_{k}\left(0\right)$$

In exponential smoothing, all previous measurements contribute to the smoothed value, but their contribution is suppressed by increasing powers of the parameter *α*. The mapping from the metric, *Q*, to the probability function, *P*, is subject to two conditions:

1. The probability assigned to a channel is an increasing function of its quality metric:
  (20) $$Q_{i}^{\prime }\left(t+1\right)\leq Q_{j}^{\prime }\left(t+1\right)\to P_{i}\left(t+1\right)\leq P_{j}\left(t+1\right)$$
  This condition results in good channels being used more often than bad channels.
2. The target average metric, ${\bar{Q}}^{\prime }\left(t+1\right)$, must be above a certain threshold *ξ*:
  (21) $$\sum_{i=1}^{K}P_{i}\left(t+1\right)\cdot Q_{i}^{\prime }\left(t+1\right)\geq \xi$$
  This condition results in robustness of the link between the nodes.
  The following mapping function fulfils the above conditions:
  (22) $$\{\begin{array}{ccc} P_{k}=(\beta +c\cdot d_{k})/\delta & if & d_{k}\geq 0\\ P_{k}=(\beta +s\cdot d_{k})/\delta & if & d_{k}\leq 0 \end{array}$$
  where the term *d_k_* is the difference between the predicted metric and the threshold *ξ*:
  (23) $$d_{k}=Q_{k}^{\prime }-\xi$$
  A positive *d_k_* value indicates a good channel and a negative value indicates a bad channel. The term *c* determines how good channels are rewarded, while the term *s* determines how bad channels are punished. The larger the *c* parameter, the more often good channels are used, and the larger the *s* parameter, the less often bad channels are used. The term *δ* is a normalizing factor that ensures $\sum P_{i}=1$:
  (24) $$\delta =\sum_{i=1}^{K_{g}}\left(\beta +c\cdot d_{i}\right)+\sum_{i=1}^{K_{b}}\left(\beta +s\cdot d_{i}\right)$$
  where *K_g_* and *K_b_* are the number of good and bad channels respectively. The term *β* is chosen so that the condition $\sum_{i=1}^{K}P_{i}(t+1)\cdot Q_{k}^{\prime }(t+1)=\xi$ is fulfilled. The case when *c* = *s* = 1 and *β* = *ξ* results in
  (25) $$P_{k}=\frac{Q_{k}^{\prime }}{\delta }=\frac{Q_{k}^{\prime }}{\sum_{i=1}^{K}Q_{i}^{\prime }}$$
  which represents the WRFH probability function.

**Figure 12:**
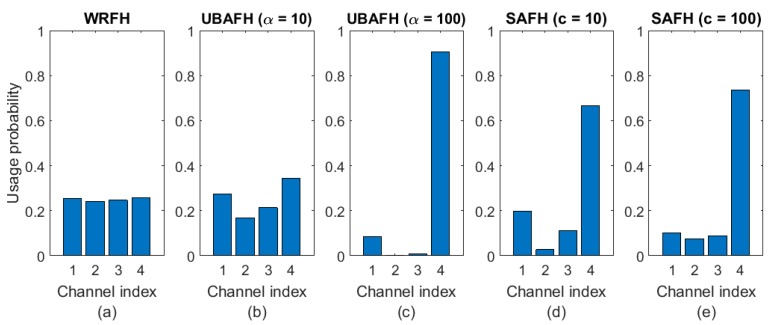
Comparison of channel usage between different frequency hopping techniques: (**a**) WRFH; (**b**) UBAFH with α=10; (**c**) UBAFH with α=100; (**d**) SAFH with c=10; (**e**) SAFH with c=100.

As an illustrative example, let us consider a four-channel problem with *Q*: {0.84, 0.8, 0.82, 0.86}. Table 1 shows the channel usage probabilities for the WRFH, UBAFH and SAFH techniques for the specified parameter values. Same results are represented in Figure 12. It can be observed how increasing the temperature parameter in the UBAFH technique penalizes the usage of the worst channels in favour of the best ones. The same happens when increasing the *c* parameter in the SAFH technique.

## 6. Method Description

This section describes the method that analyses the quality of the links between nodes in a Wireless Sensor Network (WSN) in the presence of other coexisting networks. In Section 4 we introduced some metrics that can be used to characterize and determine the quality of the available frequency channels. In Section 5 we described different frequency hopping techniques which select a subset of channels and define a hopping sequence, avoiding channels with lower quality metrics to a greater or a lesser extent depending on the hopping technique. These quality metrics and hopping techniques are interlaced and compared to find the best subset of channels and hopping sequence that result in a minimum number of interferences, improving network throughput, reliability and resilience against security attacks.

The proposed method can be divided in the following steps:

1. Evaluation of the interfering environment.
2. Frequency channel selection.
3. Packet Error Rate (PER) calculation.
4. Determination of the topology.

In the first step, the interfering environment is evaluated by determining the noise coming from coexisting networks. The interfering noise is evaluated at every point of interest, that is, at every location of the nodes of the WSN under study. Over a specified observation time, the RSSI values are measured for each of the centre frequencies of the available channels in the WSN of interest. From this RSSI values, all the quality metrics of Section 4 are calculated. Figure 2 showed an example of RSSI measurements for an observation time of 1 s and for the 16 channels available in a IEEE 802.15.4 network, from which the statistical properties of Figure 4 were calculated.

**Figure 13:**
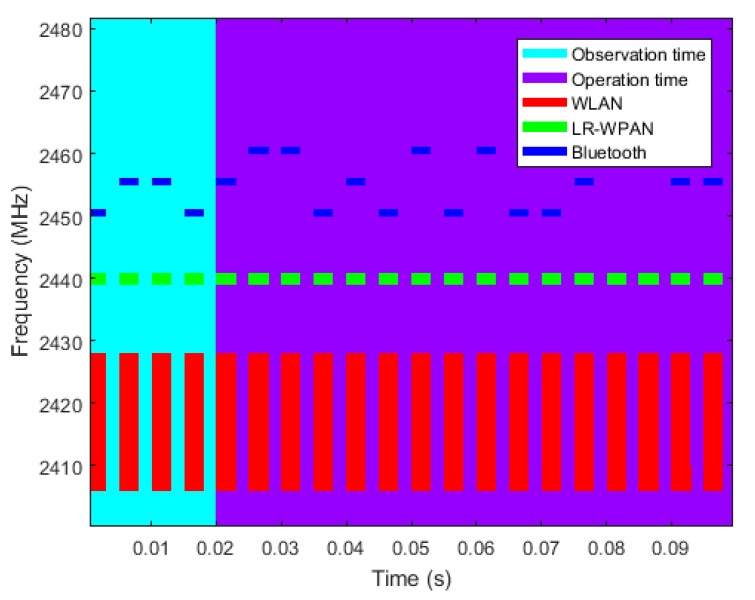
Representation of observation (blue) and operation (purple) times and example of channel occupancy with two static networks (WLAN and LR-WPAN) and a dynamic network (Bluetooth) that hops over three frequency channels. The coexisting networks will be analysed during the observation time to determine the operation of the network of interest during the operation time.

In the next step, frequency channel selection, the optimal subset of channels is calculated for each of the available quality metrics of Section 4 and hopping techniques of Section 5. The quality metrics are normalized according to Equations (3) and (4) to obtain a normalized channel gain, *H*, that can be used for every hopping technique of Section 5. The result will be a *nxm* matrix containing a subset of channels for each combination of quality metric and hopping technique, *n* being the number of employed quality metrics and *m* the number of employed hopping techniques. Along with the subset of channels, the hopping sequence is also calculated, that is, the frequency to be used every time the system hops from one channel to another. The obtained hopping sequence will be used by the WSN of interest for a specified operation time. Figure 13 shows the observation time during which the interfering environment is evaluated and the operation time during which the WSN of interest operates. An example of channel occupancy for WLAN, LR-WPAN and Bluetooth networks is also represented. WLAN and LR-WPAN networks stay in static channels with 22 MHz and 3 MHz bandwidth respectively. In order to simplify the example, the Bluetooth network hops over only three channels with 1 MHz bandwidth. 

Once the interfering environment is evaluated and the best frequency sequence is determined, the Packet Error Rate (PER) is calculated for every possible bidirectional connection between nodes of the WSN of interest. For an error to occur in the communication between two nodes, the signal of interest and the interfering noise must coexist in time and frequency, and the Signal to Noise Ratio (SNR) must be higher than the receiving sensitivity. The propagation of the signal of interest is modelled according to the free-space path loss model:

(26) $$P_{d}=P_{0}\cdot \left(\frac{4\pi df}{c}\right)$$

where $P_{o}$ is the transmission power, f the signal frequency, c the speed of light and $P_{d}$ the received power at distance d.

**Figure 14:**
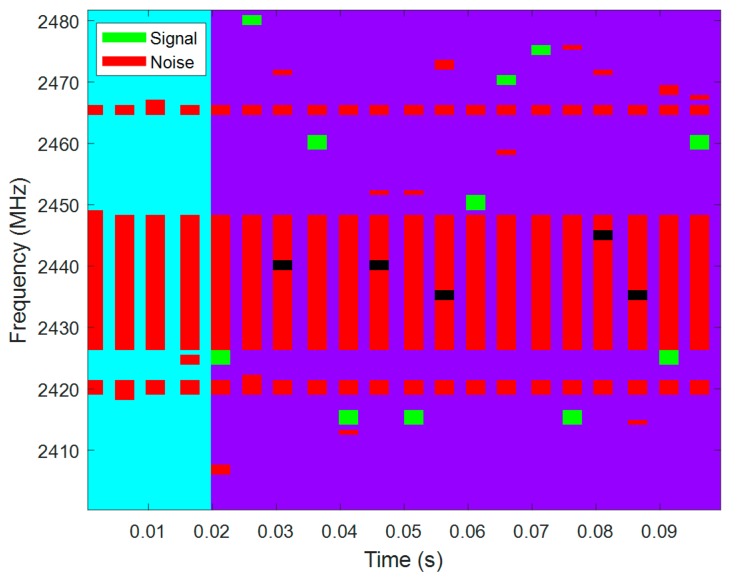
Signal (green) and noise (red) overlapping. There are different sources that contribute to noise: static networks and dynamic networks that hop over different frequencies. The noise is analysed during the observation time (blue) to determine the hopping sequence of the signal of interest. The signal of interest is only transmitted during the operation time (purple). The overlapping of signal and noise is represented in black. This overlap only implies time and frequency coexistence; for an error to occur, the SNR must be higher than the receiving sensitivity of the nodes too.

The overlapping of the signal of interest and the interference noise can be seen in Figure 14. The signal of interest (between two specific nodes) is represented in green, starting after the observation time and hopping from channel to channel within the operation time. All the contributions from different coexisting networks to the interference noise are represented in red, and the overlaps between signal and noise are represented in black.

**Figure 15:**
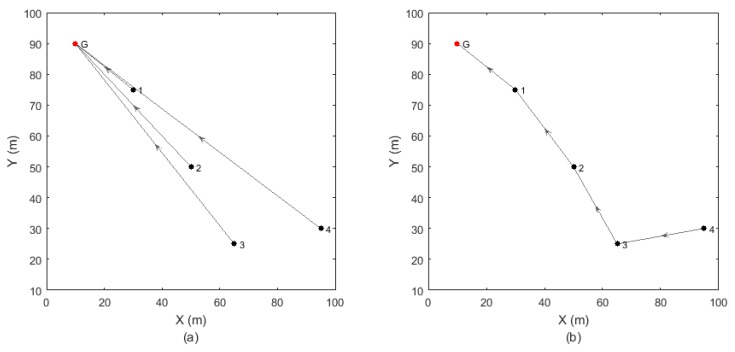
Different network topologies: (**a**) Single-hop: each node (black dots) communicates directly to the gateway (red dot); (**b**) Multi-hop: the nodes communicate to the gateway hopping through other nodes.

In the last step, determination of the topology, the topology of the WSN can be determined taking into consideration the distances between nodes and the resulting overall PER. Figure 15 shows two types of topologies where each node communicates directly to the gateway (single-hop topology) or through other nodes (multi-hop topology). When a node communicates to the gateway through other nodes, the PERs of all the transmissions are multiplied to obtain the overall PER.

In terms of energy consumption, it might be preferable to hop among different nodes, so that the communication distances are reduced and the transmission power of the nodes can be lowered. In return, this may involve increasing the overall PER. On the contrary, to minimize the overall PER, it is most likely that each node has to communicate directly to the gateway, but in exchange for increasing the transmission distances and the consequent power consumption. Assigning different weights to the overall PER and the maximum transmission distance may result in different topologies, and will help to choose the topology with the best equilibrium between overall PER and energy consumption.

## 7. Evaluation

The evaluation of the presented method is performed beginning with a simulated interfering environment. It has been done so to have an illustrative interfering noise, coming from both static and dynamic (frequency changing) networks, and with significant RSSI values. Even though the same analysis could have been done starting from real RSSI measurements of any specific scenario.

**Figure 16:**
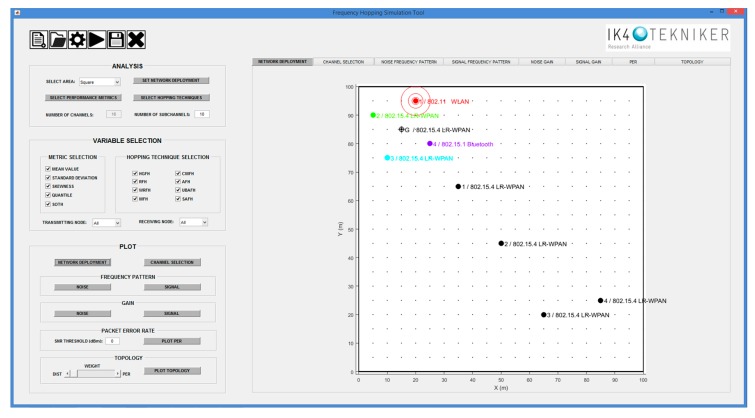
Main interface of the simulation tool. The left panels contain buttons to configure the analysis parameters and select the different plotting options. The right panel is reserved for the graphical representation of the results. The different type of results are represented in their corresponding tab within the graph panel.

To help in the evaluation of the method, a simulation tool has been developed using Matlab. The developed tool allows to define the interfering environment, deploy the network under study and graphically visualize the results of the calculations. Figure 16 shows the main window of the developed simulation tool.

**Figure 17:**
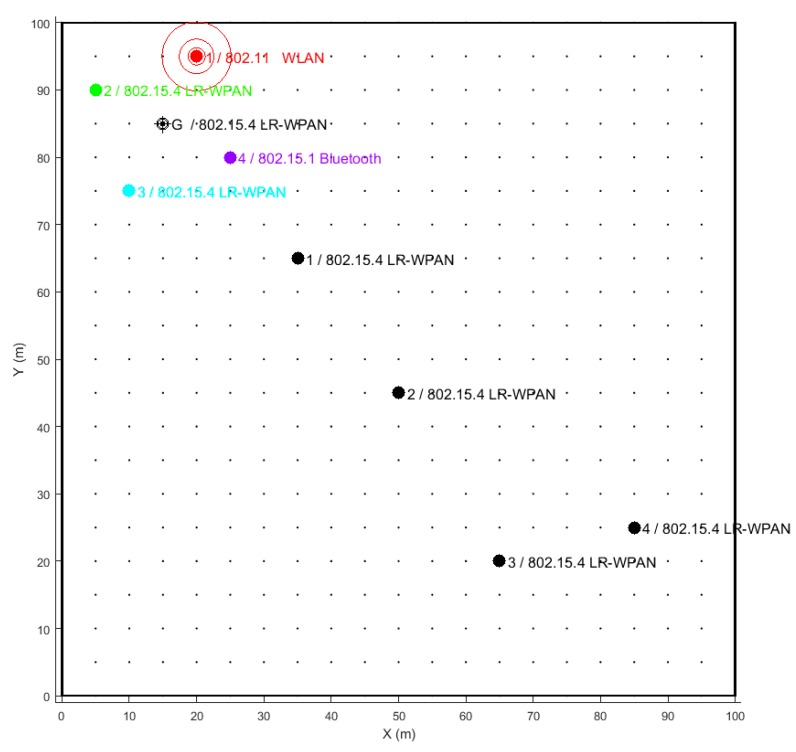
Example of an 802.15.4 network deployment with four nodes (black dots) and a gateway (G). The gateway is surrounded by four interfering networks (coloured markers).

The interfering environment is simulated by placing potential coexisting networks close to the network of interest. The simulation tool allows to choose between different standards: IEEE 802.11, IEEE 802.15.1 and IEEE 802.15.4. As an illustrative example, let us consider the deployment of an IEEE 802.15.4 network as represented in Figure 17, consisting of four sensor nodes (black dots) that have to communicate with a gateway (G). The network has to coexist with one IEEE 802.11 (WLAN), two IEEE 802.15.4 (LR-WPAN A and LR-WPAN B) and one IEEE 802.15.1 (Bluetooth) networks (coloured markers) located close to the gateway. Our IEEE 802.15.4 network has the ability to hop among the available 16 channels. 

From the transmission power of each interfering network, the interference noise is calculated at every location of the nodes of the network under study. The signal propagation is modelled according to the free-space path loss (Equation (26)). The transmission powers of the interfering networks are shown in Table 2, along with the transmission power and the receiving sensitivity of the network of interest.

The developed tool allows to configure the observation and operation times, as well as the hopping time and the slot times of each network. For the reduced hop set techniques, the number of channels to employ must be defined as well. Table 3 summarizes the time parameters employed in the evaluation example. The interference noise is observed for 0.1 s and, then, the connections of the network of interest are evaluated for an operation time of 0.9 s. Therefore, the total analysis time results in 1 s, with a time resolution (time step) of 1 ms. The slot and wait times of all the networks are set to the same values: 3 ms and 2 ms respectively. For the WSN of interest and the interfering Bluetooth network, the hop times are set to 5 ms, meaning that every 5 ms these networks hop from one frequency channel to another.

**Figure 18:**
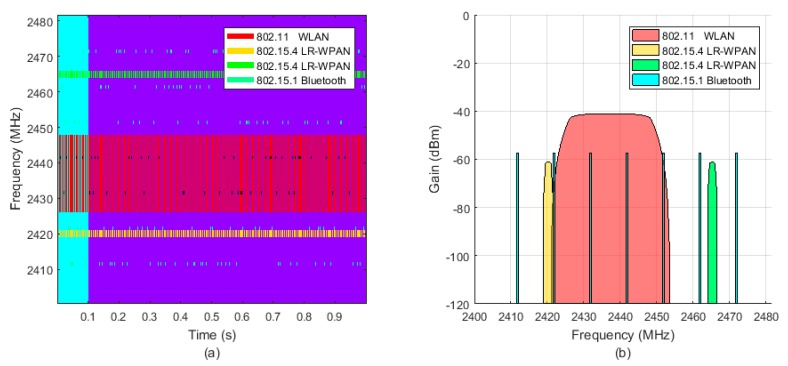
Interference noise at the gateway location: (**a**) Frequency pattern. The WLAN network transmits in the 6th channel of the 802.11 standard, centred at 2437 MHz with a bandwidth of 22 MHz (red). The LR-WPAN A network transmits in the 4th channel of the 802.15.4 standard, centred at 2420 MHz with a bandwidth of 3 MHz (yellow). The LR-WPAN B network transmits in the 13th channel, centred at 2465 MHz (green). The Bluetooth network transmits over seven frequency channels with a bandwidth of 1 MHz each, hopping from channel to channel in a random way (blue); (**b**) Signal strength. The signal strength of each interfering network at the gateway position is calculated according to the free-space path loss model. The four interfering networks are at the same distance from the gateway, but have different signal strengths as they have different transmission powers.

The IEEE 802.11 (WLAN) and the interfering IEEE 802.15.4 (LR-WPAN A and LR-WPAN B) networks transmit in static frequency channels, while the IEEE 802.15.1 (Bluetooth) network hops from frequency to frequency in a random way. For simplicity, a reduced set of seven channels have been selected from the available 79 of the Bluetooth network. Figure 18 shows the occupancy of the 2.4 GHz bandwidth over time and the amplitude of the interference noise as received by the gateway.

**Figure 19:**
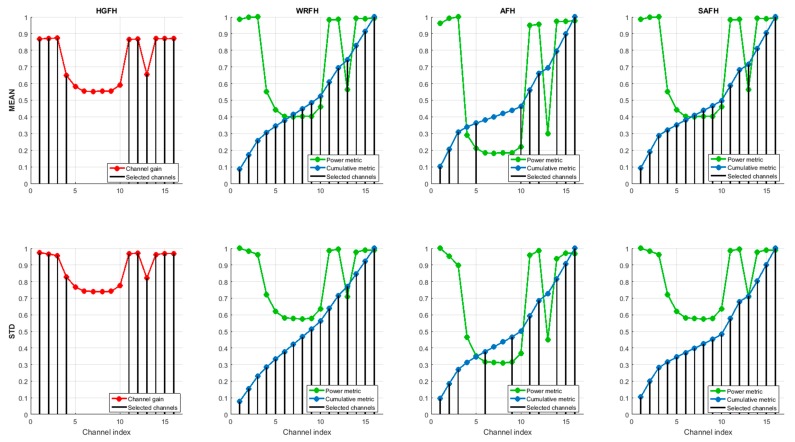
Channel selection. HGFH technique directly uses the channel gain (red) to select the 10 best channels. WRFH, AFH and SAFH techniques use the cumulative metric (blue) derived from the power metric (green) to select the best channels. In all the case, the selected channels are represented as vertical black lines.

Figure 19 represents the channel selection for different metrics and hopping techniques. In order to simplify the visualization, only four hopping techniques (HGFH, WRFH, AFH and SAFH) and two metrics (mean value and standard deviation of RSSI) have been represented, but the analysis has been done for all the eight hopping techniques in Section 5 and all the five metrics in Section 4, as will be summarized at the end of this section. The configuration parameters of some of the metrics and hopping techniques (when needed), are presented in Table 4.

For the reduced-hop-set techniques (HGFH and AFH), M=10 channels have been selected. The HGFH technique selects the 10 best channels directly from the channel gain, while the AFH technique selects the 10 best channels from the cumulative sum of the modified power metric (Equation (11)). With HGFH, both the mean and standard deviation metrics result in the same subset of channels, whereas with AFH, the resulting subset of channels are slightly different. The WRFH and the SAFH use all the available channels, but the best channels are used more often than the worst ones. The channel quality is determined from the cumulative metric: the higher the slope of the cumulative metric, the higher the probability for the corresponding channel to be selected.

**Figure 20:**
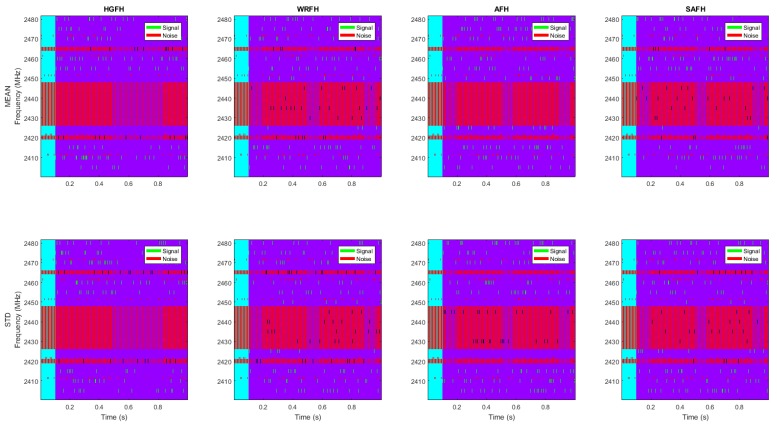
Frequency pattern of signal and noise for different hopping techniques and quality metrics. The interfering noise is represented in red and the signal of interest in green. The same noise pattern is considered for all the hopping techniques and all the quality metrics. Different hopping techniques and different quality metrics result in different frequency patterns for the signal of interest. The overlapping of the noise and signal of interest is represented in black.

The resulting hopping sequence is represented in Figure 20. The interference noise from all the interfering networks is represented in red and the signal of interest is represented in green. The overlapping of the interference noise and signal is represented in black, but as we have mentioned before, aside from the overlapping, the signal to noise ratio has to be higher than the receiving sensitivity for the communications to fail. The bad channel avoidance is best observed here. The HGFH, for instance, totally avoids the 22 MHz band of the WLAN network. However, it does not manage to avoid the 3 MHz bands of the LR-WPAN A (channel 4) and LR_WPAN B (channel 13) networks when choosing 10 channels. The AFH tries to avoid the occupied channels while maintaining certain level of frequency dispersion and, thus, the WLAN is not always avoided. WRFH and SAFH do not avoid any of the occupied channels, but channels within the 22 MHz band of the WLAN network are used less often than good channels.

**Figure 21:**
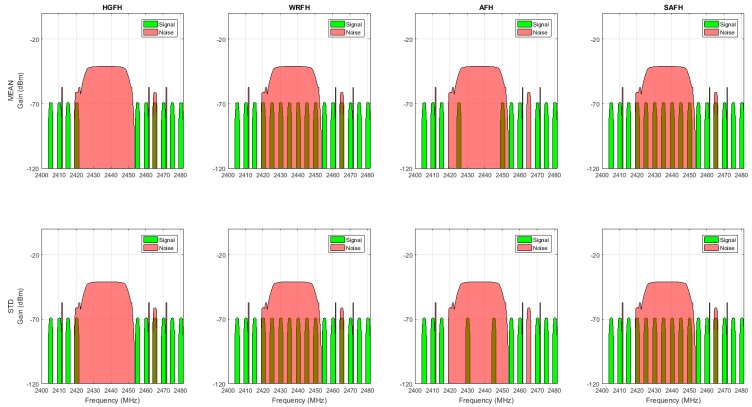
Strength of signal and noise for different hopping techniques and quality metrics. The interfering noise is represented in red and the signal of interest in green. The noise is the sum of all the interfering networks as received at the gateway. The signal of interest is the signal strength received at the gateway coming from the first node.

The amplitude gains of the signal of interest and noise are represented in Figure 21 for the specific connection between the first node and the gateway. The noise corresponds to the signal strength of all the contributing interference networks received at the gateway position, and the signal of interest corresponds to the signal strength received at the gateway coming only from the first node. For the considered transmission powers, if signal and noise overlap in time and frequency, the communication will fail as the strength of the signal of interest is lower than the noise at the gateway. The frequency pattern of Figure 20 will be the same for all the nodes in the network, but the amplitude of the different signals will depend on the distance between the nodes and between the nodes and the interfering networks.

**Figure 22:**
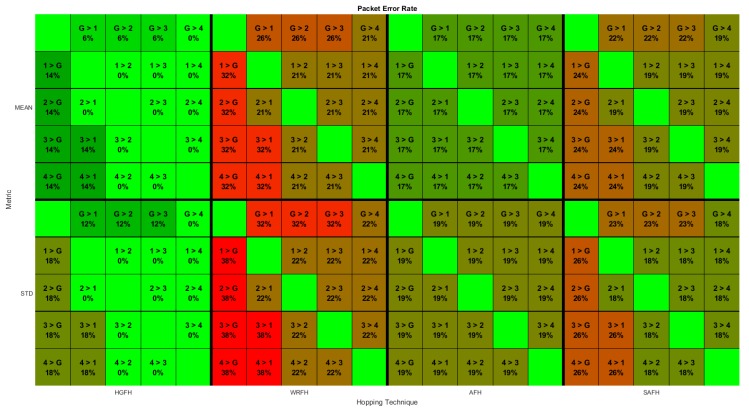
Packet Error Rate for all possible node connections for different quality metrics and hopping techniques. Each element contains the Packet Error Rate (PER) value (percentage) for the specified connection. 1>G, for instance, indicates the connection from node 1 to the gateway.

For each possible bidirectional connection between nodes of the network under study, the PER is calculated from the number of interferences during the operation time. The result will be a matrix for each quality metric and hopping technique as represented in Figure 22. 

**Figure 23:**
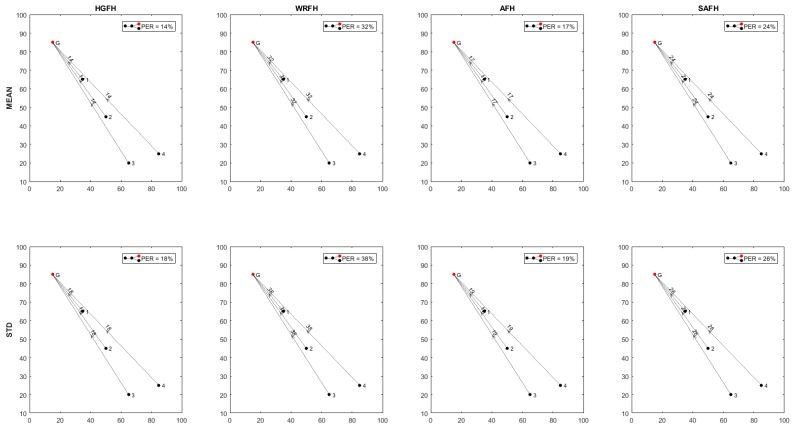
Inferred topologies with $w_{PER}=1$ and $w_{DIST}=0$. Minimizing the overall PER results in direct connections between nodes and gateway for all the hopping techniques and quality metrics.

**Figure 24:**
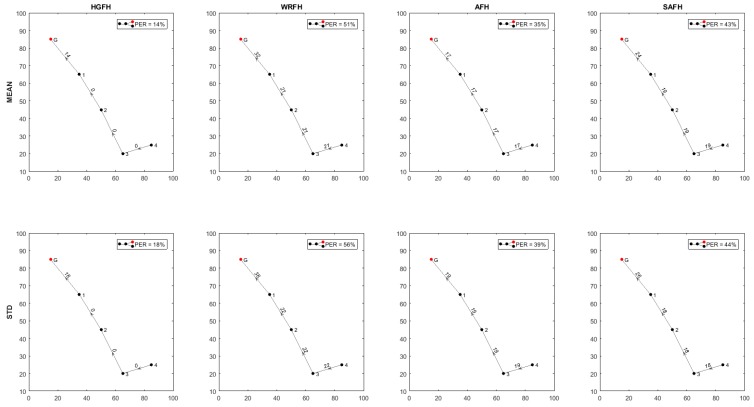
Inferred topologies with $w_{PER}=0$ and $w_{DIST}=1$. Minimizing the transmission distances results in multi-hop connections for all the hopping techniques and quality metrics. The overall PER is considerably increased, except for the HGFH technique, which remains the same as with single-hop topology.

**Figure 25:**
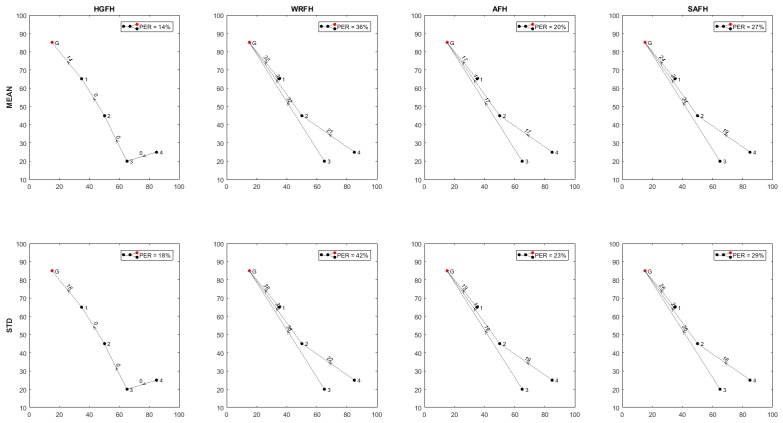
Inferred topologies with $w_{PER}=0.5$ and $w_{DIST}=0.5$. Different hopping techniques and different quality metrics result in different topologies.

From the PER of each connection, the topology of the network can be determined in terms of overall packet error rate and transmission distances. Figure 23, Figure 24 and Figure 25 show the resulting topologies when assigning different weights to the transmission distances and the resulting PER. Only attending to the overall PER results in direct connections between the nodes and the gateway (Figure 23), while minimizing the transmission distances results in multi hop transmissions (Figure 24). In both cases, the same topologies result for all the quality metrics and hopping techniques. When assigning equal weights to the transmission distances and the overall PER, different topologies result for different quality metrics and hopping techniques (Figure 25). With the HGFH, for instance, hopping from node to node in order to minimize the transmission distances does not penalize the overall PER at all, while with the other hopping techniques, the overall PER is considerably increased as the transmission distances are reduced.

Table 5 summarizes the overall PER results for all the quality metrics in Section 4 and all the hopping techniques in Section 5 for equal weights of overall PER and transmission distances. For most of the considered metrics, the RFH results to be the worst hopping technique as we could expect. The WRFH technique significantly improves the performance of the RFH, but is still far from the performance level of the other hopping techniques. Among the other hopping techniques, it cannot be said that a specific technique is much better than the others, and the same happens with the quality metrics. For the considered scenario, the combination of UBAFH technique and 95% quantile metric results to be the best solution with a 9% of overall PER, but different interfering scenarios might result in other quality metrics and hopping techniques being the best. Configuration parameters other than the ones presented in Table 4 could also yield a different solution. Nevertheless, the main objective of the method here presented is not to determine a quality metric and a hopping technique that best fits most of the possible scenarios, but to automatically find the best solution for any specific scenario after analysing all the possible solutions.

## 8. Conclusions

In this paper, we presented a method for selecting the best frequency hopping strategy. The proposed method leverages a set of standard frequency hopping techniques and quality metrics in order to homogenize and normalize them, so they can be fairly compared based on quality and performance indicators. The main objective of the method is to provide an interference avoidance mechanism to reduce the need for retransmissions, minimize energy consumption, avoid the unnecessary degradation of QoS and reliability as well as protect against interference based security attacks.

The method has the advantage of dynamically selecting the best channel quality metric and the best hopping technique taking into account the communication channel quality and environment interference conditions. As opposed to static and standalone solutions found in the state of the art, in case of channel interference, the method supports the selection of the best frequency hopping strategy to minimize the side effects of an interfered frequency and is able to dynamically adapt to changing conditions. The final result is a subset of frequency channels, as well as a hopping sequence, that minimizes the interference with coexisting networks and the effects of interference based security attacks.

The method is designed to be used in two complementary scenarios: (a) as a deployment planning supporting tool for the selection of the best frequency channels, channel quality metrics and frequency hopping techniques given a known interference scenario and (b) as the base for the implementation of a runtime management component for the dynamic selection of communication frequency channels under changing interference conditions.

The method is extensible and easily accommodates additional frequency hopping techniques, quality metrics and performance indicator approaches. Additionally, the method can also be extended to take into account other characteristics and restrictions of constrained systems (such as processing power or energy consumption) in order to select the most suitable solution.

A simulation tool has been developed to assist in the validation and implementation of the method. The simulation tool allows for the definition and simulation of the interfering environment, but the whole analysis is perfectly applicable to real environments. That is, the received signal strength indicator (or some other channel quality indicator) in a real environment could be measured and used as input for the method implementation, and the channel selection and the hopping sequence would be obtained from the tool.

It is left for future work to test the implementation of a runtime channel selection component based on the proposed method and its verification against the simulation tool. For that, a real deployment would have to be analysed in terms of received signal strength indicator for the interfering nodes and packet error rate for node connections in the wireless sensor network under study, and then compared with the same simulated scenario. It is also left for future research the inclusion of additional input parameters to the method to take into account other restrictions of the environment or the system, such as characteristics of constrained systems.

## Figures and Tables

**Table 1:** Comparison of channel usage for Weighted Random Frequency Hopping (WRFH), Utility Based Adaptive Frequency Hopping (UBAFH) and Smooth Adaptive Frequency Hopping (SAFH) techniques.

			WRFH	UBAFH		SAFH	
			$P_{k}=\frac{Q_{k}}{\sum_{i=1}^{K}Q_{i}}$	$P_{k}=\frac{Q_{k}^{\alpha }}{\sum_{i=1}^{K}Q_{i}^{\alpha }}$		*ξ* = 0.85 *α* = 1 *c* = 10 *s* = 1	*ξ* = 0.85 *α* = 1 *c* = 100 *s* = 1
				α=10	α=100		
$Q_{1}$	0.84	$P_{1}$	0.253	0.273	0.086	0.197	0.100
$Q_{2}$	0.8	$P_{2}$	0.241	0.168	0.001	0.027	0.075
$Q_{3}$	0.82	$P_{3}$	0.247	0.214	0.008	0.111	0.088
$Q_{4}$	0.86	$P_{4}$	0.259	0.345	0.906	0.665	0.737

**Table 2:** Transmission powers and receiving sensitivities for the interfering networks and the wireless sensor network under study.

	INTERFERENCE				WSN
	WLAN	Bluetooth	LR-WPAN A	LR-WPAN B	802.15.4
TRANSMISSION POWER	100 mW	2.5 mW	1 mW	1 mW	1 mW
RECEIVING SENSITIVITY	-	-	-	-	−90 dBm

**Table 3:** Time parameters employed in the analysis.

**OBSERVATION TIME**	0.1 s				
**OPERATION TIME**	0.9 s				
**ANALYSIS TIME**	1 s				
**TIME STEP**	1 ms				
	**INTERFERENCE**				**WSN**
	**WLAN**	**Bluetooth**	**LR_WPAN A**	**LR_WPAN B**	**802.15.4**
**SLOT TIME**	3 ms	3 ms	3 ms	3 ms	3 ms
**WAIT TIME**	2 ms	2 ms	2 ms	2 ms	2 ms
**HOP TIME**	-	5 ms	-	-	5 ms

**Table 4:** Configuration parameters for quality metrics and hopping techniques.

**QUALITY METRIC PARAMETERS**			
QUANTILE		SOTH	
95%		th=−60 dBm	
**HOPPING TECHNIQUE PARAMETERS**			
CMFH	AFH	UBAFH	SAFH
*ξ* = 0.1	*α* = 0.5	*α* = 2	*ξ* = 0.85
			*α* = 1
			*c* = 10
			*s* = 1

**Table 5:** Overall PER with $w_{PER}=0.5$ and $w_{DIST}=0.5$.

		HOPPING TECHNIQUES							
		HGFH	RFH	WRFH	MFH	CMFH	AFH	UBAFH	SAFH
quality metrics	mean	14%	51%	36%	22%	19%	20%	17%	27%
	std	18%	52%	42%	48%	49%	23%	24%	29%
	skewness	19%	50%	45%	57%	43%	53%	41%	15%
	95% quantile	18%	42%	19%	23%	19%	19%	9%	24%
	soth (−60 dBm)	22%	49%	31%	35%	18%	20%	21%	33%
